# Is Host Filtering the Main Driver of Phylosymbiosis across the Tree of Life?

**DOI:** 10.1128/mSystems.00097-18

**Published:** 2018-10-23

**Authors:** Florent Mazel, Katherine M. Davis, Andrew Loudon, Waldan K. Kwong, Mathieu Groussin, Laura Wegener Parfrey

**Affiliations:** a Department of Botany, University of British Columbia, Vancouver, British Columbia, Canada; b Biodiversity Research Centre, University of British Columbia, Vancouver, British Columbia, Canada; c Department of Zoology, University of British Columbia, Vancouver, British Columbia, Canada; d Center for Microbiome Informatics and Therapeutics, Massachusetts Institute of Technology, Cambridge, Massachusetts, USA; e Department of Biological Engineering, Massachusetts Institute of Technology, Cambridge, Massachusetts, USA; University of California, Riverside

**Keywords:** endosphere, gut microbiome, holobiont, macroevolution, plant microbiome, rhizosphere

## Abstract

Host-associated microbiota composition can be conserved over evolutionary time scales. Indeed, closely related species often host similar microbiota; i.e., the composition of their microbiota harbors a phylogenetic signal, a pattern sometimes referred to as “phylosymbiosis.” Elucidating the origins of this pattern is important to better understand microbiota ecology and evolution. However, this is hampered by our lack of theoretical expectations and a comprehensive overview of phylosymbiosis prevalence in nature. Here, we use simulations to provide a simple expectation for when we should expect this pattern to occur and then review the literature to document the prevalence and strength of phylosymbiosis across the host tree of life. We demonstrate that phylosymbiosis can readily emerge from a simple ecological filtering process, whereby a given host trait (e.g., gut pH) that varies with host phylogeny (i.e., harbors a phylogenetic signal) filters preadapted microbes. We found marked differences between methods used to detect phylosymbiosis, so we proposed a series of practical recommendations based on using multiple best-performing approaches. Importantly, we found that, while the prevalence of phylosymbiosis is mixed in nature, it appears to be stronger for microbiotas living in internal host compartments (e.g., the gut) than those living in external compartments (e.g., the rhizosphere). We show that phylosymbiosis can theoretically emerge without any intimate, long-term coevolutionary mechanisms and that most phylosymbiosis patterns observed in nature are compatible with a simple ecological process. Deviations from baseline ecological expectations might be used to further explore more complex hypotheses, such as codiversification.

**IMPORTANCE** Phylosymbiosis is a pattern defined as the tendency of closely related species to host microbiota whose compositions resemble each other more than host species drawn at random from the same tree. Understanding the mechanisms behind phylosymbiosis is important because it can shed light on rules governing the assembly of host-associated microbiotas and, potentially, their coevolutionary dynamics with hosts. For example, is phylosymbiosis a result of coevolution, or can it be generated by simple ecological filtering processes? Beyond qualitative theoretical models, quantitative theoretical expectations can provide new insights. For example, deviations from a simple baseline of ecological filtering may be used to test more-complex hypotheses (e.g., coevolution). Here, we use simulations to provide evidence that simple host-related ecological filtering can readily generate phylosymbiosis, and we contrast these predictions with real-world data. We find that while phylosymbiosis is widespread in nature, phylosymbiosis patterns are compatible with a simple ecological model in the majority of taxa. Internal compartments of hosts, such as the animal gut, often display stronger phylosymbiosis than expected from a purely ecological filtering process, suggesting that other mechanisms are also involved.

## Abstract (precis)

Phylosymbiosis is a pattern defined as the tendency of closely related species to host microbiota whose compositions resemble each other more than host species drawn at random from the same tree. Understanding the mechanisms behind phylosymbiosis is important because it can shed light on rules governing the assembly of host-associated microbiotas and, potentially, their coevolutionary dynamics with hosts. For example, is phylosymbiosis a result of coevolution, or can it be generated by simple ecological filtering processes? Beyond qualitative theoretical models, quantitative theoretical expectations can provide new insights. For example, deviations from a simple baseline of ecological filtering may be used to test more-complex hypotheses (e.g., coevolution). Here, we use simulations to provide evidence that simple host-related ecological filtering can readily generate phylosymbiosis, and we contrast these predictions with real-world data. We find that while phylosymbiosis is widespread in nature, phylosymbiosis patterns are compatible with a simple ecological model in the majority of taxa. Internal compartments of hosts, such as the animal gut, often display stronger phylosymbiosis than expected from a purely ecological filtering process, suggesting that other mechanisms are also involved.

## INTRODUCTION

Rich communities of microorganisms, collectively called the microbiota, make their living in and on macro-organisms. Within a host species, microbiota compositions can vary dramatically across body sites (1), across individuals that reside in different environments (2), or across host phenotypic traits, such as age (3) or diet (4). Although some previous studies have detected a few heritable microbial taxa (5), recent work suggests that host genetics plays a minor role in shaping the human gut microbiota and that the cumulative abundance of heritable microbes appears to be very low (2). Thus, microbiota composition can be highly dynamic and variable over short time scales in response to diet and/or environmental factors, such as pathogen infection (6). However, the variability of microbiota compositions during this short time scale (within species) is often less than long-term variability (between species). Indeed, one of the most intriguing patterns of host-associated microbiotas is that they are often host species specific (i.e., individuals within a host species harbor more similar microbiota than individuals from two different host species) and that closely related host species harbor similar microbiotas. In other words, microbiota composition harbors some degree of phylogenetic signal, defined as “the tendency of related species to resemble each other more than species drawn at random from the same tree” (7, 8). The phylogenetic signal in host-associated microbiota is now often referred to as “phylosymbiosis” (9, 10). As with phylogenetic signal, phylosymbiosis strength can be quantified with different metrics, and its significance can be assessed by comparing the observed metric values to appropriate null expectations (e.g., via randomization of the data). Here, we use the term “phylosymbiosis” without assuming any underlying process (11) and consider the word “symbiosis” in its widest sense, denoting the simple cooccurrence of microbes and hosts (12). From here on, we will focus on the interspecific component of phylosymbiosis, assuming that microbiotas are host species specific (i.e., individuals within a host species harbor more similar microbiota than individuals from two different host species).

Understanding the importance and implications of phylosymbiosis is hampered by our lack of theoretical expectations and a comprehensive overview of its prevalence in nature. Only qualitative theoretical models for when we can expect phylosymbiosis have been proposed (13). For example, one could suggest that long-term coevolution between gut microbes and their hosts is driving phylosymbiosis. This model implies a long-term and intimate association between hosts and their microbes (13), as illustrated in the textbook example of the symbiosis between *Buchnera* and their insect hosts, the pea aphids (14). It is important to recognize that the case of *Buchnera* consists of one specific lineage of bacteria that has coevolved with its hosts, whereas phylosymbiosis examines the pattern of entire microbial communities and their relationship to host phylogeny. Understanding the mechanistic origins of phylosymbiosis has stimulated much debate, and coevolution is unlikely to be fully responsible for driving phylosymbiosis (11, 13, 15, 16). In particular, Moran and Sloan (13) argued that phylosymbiosis can be the product of a simple ecological filtering model. This model posits that a host trait selectively filters adapted microbes from the environment; for example, a particular gut pH (17) or oxidative state may select for particular combinations of microbes that are adapted to these conditions. If the host trait controlling microbial assembly harbors some degree of phylogenetic signal, then we can also expect microbiota compositions to show phylosymbiosis (15, 18, 19). To our knowledge, this qualitative prediction has never been verified using explicit ecological simulations. A quantitative theoretical expectation for when we expect phylosymbiosis to happen will be valuable, as deviations from this simple ecological baseline could be used to test more-complex hypotheses (e.g., codiversification).

Here, our aims are (i) to provide a simple quantitative ecological baseline for when we should expect phylosymbiosis to arise from ecological filtering, (ii) to assess the specificity and sensitivity (i.e., statistical power) of the proposed phylosymbiosis approaches to detect ecological filtering, (iii) to document phylosymbiosis prevalence across diverse host species, and (iv) to compare expected and observed patterns and discuss the potential processes shaping host-associated microbiotas. Overall, we show that phylosymbiosis is widespread but often weak, that it has been measured in a variety of ways, that it can emerge from simple ecological processes, and that the phylogenetic signal of the host trait strongly determines the strength of phylosymbiosis. Finally, the proposed methods to detect phylosymbiosis differ markedly in their statistical powers, so that future studies should aim to use similar and rigorous methods.

## RESULTS

### Theoretical expectations.

**FIG 1:**
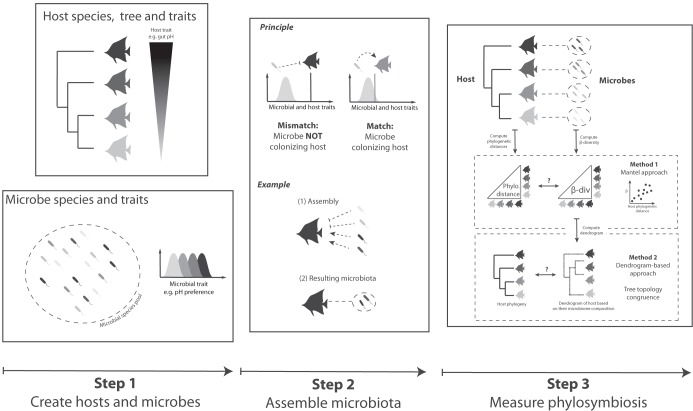
Overview of the simulation workflow. Each simulation was run in three steps. First, host and microbial species and traits were independently simulated. Second, microbiotas were assembled for each host independently by following a set of ecological rules. Assembly was either neutral, a pure ecological filtering process (as depicted in the figure) based on the match between host trait (e.g., gut pH) and microbial trait (e.g., pH preference), or a mix of ecological filtering and competition. Third, phylosymbiosis was measured using two alternative methods: one using the Mantel test (Mantel approach), the other using a topological distance metric between the host species tree and the clustering tree of microbiota compositional dissimilarities (dendrogram-based approach).

We use simulations to provide a simple quantitative ecological baseline for when we should expect phylosymbiosis to arise from ecological filtering. Simultaneously, we assess the specificity and sensitivity (i.e., statistical power) of the existing, widely used, statistical approaches that measure phylosymbiosis to detect host filtering correlated with host phylogeny (Fig. 1). We first simulate multiple host-associated microbiota sets under various ecological assembly rules and then measure phylosymbiosis using the two most common methods: Mantel and dendrogram-based approaches. The Mantel approach is based on the Mantel test (20) that measures correlation between two distance matrices, e.g., that of host phylogenetic divergences and that of their microbial community dissimilarities. The dendrogram approach (9) measures congruence between tree representations of host relatedness (e.g., with molecular phylogenies) and tree representations of microbiota similarity (e.g., hierarchical clustering tree of microbial community similarities).

We first simulate sets of microbiotas that are neutrally assembled to establish a baseline. Microbes are associated with a given host species by randomly drawing them from a microbe pool so that we do not expect microbiota compositions to recapitulate host phylogeny. We find that, as expected, microbial compositions are not significantly related to host phylogeny in most cases (see Fig. S1 in the supplemental material). Adequate statistical procedures should have high specificity (i.e., a low type I error rate), meaning a high chance of detecting true negatives. The threshold for type I error rate corresponds to the threshold chosen to declare significance in individual tests (e.g., *m *=* *5%); here, this translates to detecting correlations in 5% of the neutral simulations despite a lack of true signal. We find that the Mantel and the dendrogram-based approaches report false-positive correlations in ∼5% of neutral simulations (Fig. S1). This means that these approaches have adequate specificity to qualify as a valid statistical test (Fig. S1) to detect ecological filtering.

10.1128/mSystems.00097-18.1 FIG S1 Theoretical expectation of the significance and strength of the correlation between microbiota composition and host phylogeny (i.e., phylosymbiosis) or host trait for varying strength of simulated ecological filtering. The correlation between microbiota composition and host phylogeny (i.e., phylosymbiosis) is measured with two methods (the dendrogram-based method and the Mantel method [yellow and blue curves and boxplots, respectively]), while the correlation between microbiota composition and host trait is measured with a Mantel test (green curve and boxplots). Note that because microbiota have been directly filtered with the host trait, we expect the correlation between microbiota composition and this host trait to be higher than with host phylogeny (i.e., phylosymbiosis approaches) which imperfectly reflects the host trait. For phylosymbiosis (dendrogram-based and Mantel methods) the plots depict the relationship between the proportion of phylosymbiosis (i.e., the proportion of simulations where the phylosymbiosis metric was significantly different from null expectations, *y* axis, top plots) or the strength of the phylosymbiosis as quantified by Pearson’s *r* or Robinson-Foulds metric (*y* axis, bottom plots) and the phylogenetic signal of the host trait that filters microbes to assemble microbiotas (*x* axis). Note that Pearson’s *r* and the normalized Robinson-Foulds metric should be interpreted differently with respect to the strength of phylosymbiosis; normalized Robinson-Foulds metric values close to 0 indicate strong phylosymbiosis, while Pearson’s *r* values near 1 indicate strong phylosymbiosis. For the correlation between microbiota composition and host trait (green curves and boxplots), the plots depict the relationship between the proportion of significant Mantel tests (*y* axis, top plots) or the strength of the signal as quantified by Pearson’s *r* (*y* axis, bottom plots) and the phylogenetic signal of the host trait that filters microbes to assemble microbiotas (*x* axis). The strength of the simulated ecological filtering increases from the left to right panels: minimum for the left panels (i.e., neutral, beta.env = 0) and maximum for the right panels (beta.env = 1). These results are based on 1,000 simulation sets, each set containing 150 microbial species and 25 host species. Beta-diversity is quantified using the weighted UniFrac metric. Download FIG S1, EPS file, 2.3 MB. Copyright © 2018 Mazel et al. 2018 Mazel et al. This content is distributed under the terms of the Creative Commons Attribution 4.0 International license.

**FIG 2:**
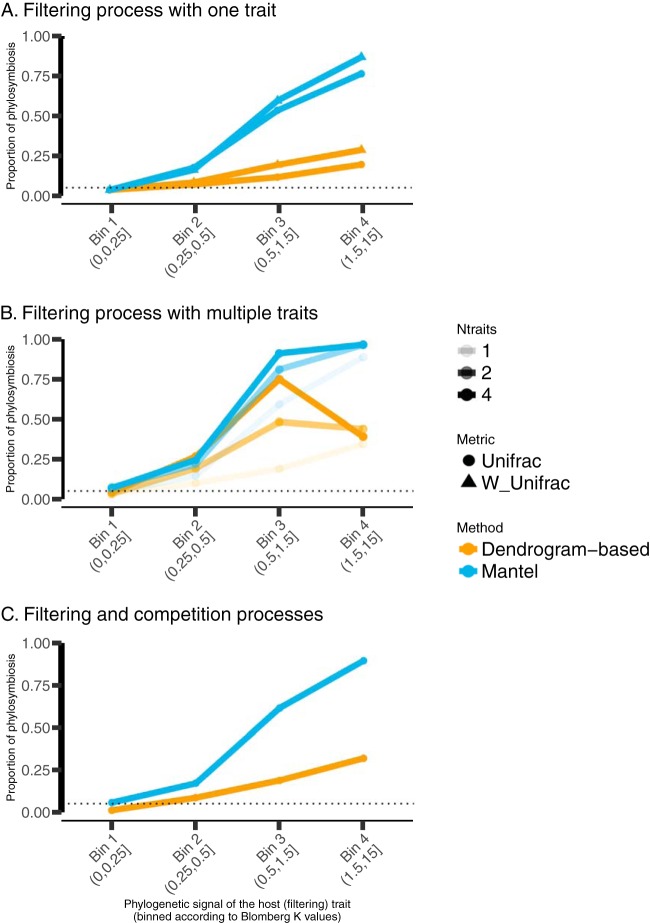
Theoretical expectation of phylosymbiosis prevalence based on simple ecological filtering. The plots depict the relationship between the phylogenetic signal of the host trait that filters microbes to assemble microbiotas (simple ecological process) and the proportion of phylosymbiosis detected (i.e., the proportion of simulations where the phylosymbiosis metric was significantly different from null expectations) in the resulting microbiotas for a single (A) or multiple (B) filtering traits and when competition and filtering are jointly assembling microbiotas (C). Note that multiple simulations have been grouped in bins of host trait phylogenetic signal (*x* axis) in order to compute the percentage of simulations that yielded phylosymbiosis. These results are based on 1,000 simulation sets in total, each set containing 150 microbial species and 25 host species. The dotted line indicates the significance threshold used in this study (0.05).

Next, we simulated a second set of microbiotas that are deterministically assembled according to filtering by a single host trait. As expected, microbial compositions are significantly correlated with this host trait (Fig. S1). This demonstrates the high sensitivity (i.e., the low type II error rate) or high chance of finding true positives (rejecting the null hypothesis among cases that do not satisfy the null hypothesis) of the Mantel test when used with the underlying ecologically determinative trait (the dendrogram-based approach cannot be used with host traits). We varied the phylogenetic signal in this host trait and assessed our ability to detect phylosymbiosis. Depending on the strength of the phylogenetic signal of the host trait, we find that phylosymbiosis ranges from strong to totally absent. When the host trait does not exhibit phylogenetic signal, both approaches detect few significant correlations (phylosymbiosis in ∼5% of runs) and thus, has high specificity, as in the neutral simulations. When the host trait exhibits phylogenetic signal, phylosymbiosis is detected with both methods in many simulations (Fig. 2A), showing that ecological filtering, when correlated with host phylogeny, can be sufficient to generate phylosymbiosis patterns.

These results also confirm that the Mantel test and the dendrogram-based approach have high sensitivity to detect host phylogenetically correlated filtering. For example, when the host trait exhibits a moderate-to-strong phylogenetic signal (0.5 to 1.5 as measured by the Blomberg K index), ∼50% (Mantel approach) and ∼20% (dendrogram-based approach) of communities have compositions that harbor phylosymbiosis (Fig. 2A). The portion of simulated communities that harbor phylosymbiosis increases to ∼90% (Mantel approach) and to ∼25% (dendrogram-based approach) when the phylogenetic signal for the host trait is very strong (Blomberg K, 1.5 to 15) (Fig. 2A). This means that the Mantel approach is more powerful than the dendrogram-based approach to detect, in our case, effects of host phylogenetically correlated filtering on microbiota compositions. Additional experiments are needed to confidently extend this conclusion to other processes shaping microbiota compositions, such as codiversification or coevolution.

**FIG 3:**
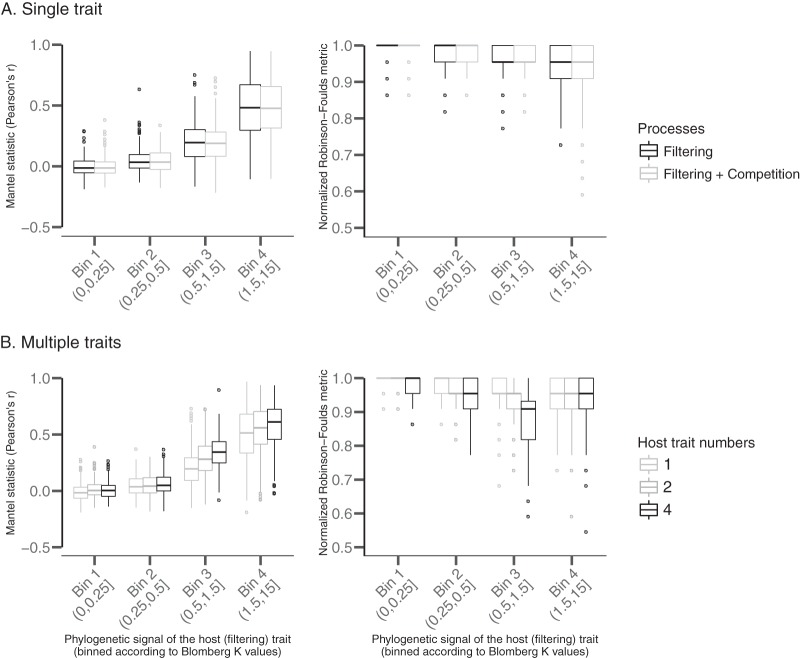
Theoretical expectation of phylosymbiosis strength based on simple ecological filtering. The plot depicts the relationship between the phylogenetic signal of the host trait that filters microbes to assemble microbiotas (pure ecological process) and the strength of phylosymbiosis as quantified by Pearson’s *r* from Mantel tests (left) or normalized Robinson-Foulds metrics (right) for one (A) or multiple (B) traits. Simulations with a single trait were run with and without microbial competition in panel A. The normalized Robinson-Foulds metric quantifies the mismatches between the topology of the microbiota dendrogram and the host tree (and varies between 0 and 1), so that a low value indicates a high congruence between topologies (i.e., strong phylosymbiosis). Pearson’s *r* from the Mantel test quantifies the correlation between host phylogenetic distances and microbiota beta-diversity (and varies between −1 and 1) so that a high positive value indicates a high correlation (i.e., strong phylosymbiosis). These results are based on 1,000 simulation sets, with each set containing 150 microbial species and 25 host species.

Significance does not assess the relative strength of the effect; a small effect can be significant with many observations. Thus, we also report the strength of phylosymbiosis as measured by the two approaches. The Mantel test quantifies strength using, for example, Pearson’s *r* between host distances and microbiota dissimilarities, while the dendrogram-based approach relies on a metric that calculates the dissimilarity of tree branching structures (with the normalized Robinson-Foulds metric, a value of 0 indicates that the two tree topologies are fully identical, and a value of 1 indicates that the two trees do not have a single bipartition in common). We find that with a moderate-to-strong phylogenetic signal (Blomberg K, 0.5 to 1.5) for the host trait, Pearson’s *r* is ∼0.2 on average. In the same range of values for the phylogenetic signal of the host trait, the normalized Robinson-Foulds metric is ∼0.92 (Fig. 3A), which suggests a weak signal for phylosymbiosis. Pearson’s *r* increases up to 0.52 as the phylogenetic signal for the host trait increases (Blomberg K > 1.5) (Fig. 3A). In contrast, the normalized Robinson-Foulds metric remains close to 0.9 regardless of the magnitude of the phylogenetic signal (Fig. 3A). Weighted UniFrac analysis performs slightly better than unweighted UniFrac analysis in detecting phylosymbiosis (compare the triangles and circles in Fig. 2A), and results hold true when using alternative beta diversity metrics (e.g., Jaccard) to measure community composition dissimilarities (Fig. S2). Importantly, our results are robust to changes in the parameters of the model used to simulate microbiota compositions and structures (Fig. S3 to S6).

10.1128/mSystems.00097-18.2 FIG S2 Theoretical expectation of the significance and strength of the correlation between microbiota composition and host phylogeny (i.e., phylosymbiosis) or host trait for varying beta-diversity metrics. The correlation between microbiota composition and host phylogeny (i.e., phylosymbiosis) is measured with two methods (the dendrogram-based method and the Mantel method [yellow and blue curves and boxplots, respectively]), while the correlation between microbiota composition and host trait is measured with a Mantel test (green curve and boxplots). Note that because microbiota have been directly filtered with the host trait, we expect the correlation between microbiota composition and this host trait to be higher than with host phylogeny (i.e., phylosymbiosis approaches), which imperfectly reflects the host trait. For phylosymbiosis (dendrogram-based and Mantel methods), the plots depict the relationship between the proportion of phylosymbiosis (i.e., the proportion of simulations where the phylosymbiosis metric was significantly different from null expectations, *y* axis, top plots) or the strength of the phylosymbiosis as a quantified by Pearson’s *r* or the Robinson-Foulds metric (*y* axis, bottom plots) and the phylogenetic signal of the host trait that filters microbes to assemble microbiotas (*x* axis). Note that Pearson’s *r* and normalized Robinson-Foulds metric should be interpreted differently with respect to the strength of phylosymbiosis; normalized Robinson-Foulds metric values close to 0 indicate strong phylosymbiosis while Pearson’s *r* values near 1 indicate strong phylosymbiosis. For the correlation between microbiota composition and host trait (green curves and boxplots), the plots depict the relationship between the proportion of significant Mantel tests (*y* axis, top plots) or the strength of the signal as a quantified by Pearson’s *r* (*y* axis, bottom plots) and the phylogenetic signal of the host trait that filters microbes to assemble microbiotas (*x* axis). The strength of the simulated ecological filtering is set to 0.5, and multiple beta-diversity metrics are tested (see the key). These results are based on 1,000 simulation sets, with each set containing 150 microbial species and 25 host species. Download FIG S2, EPS file, 2.4 MB. Copyright © 2018 Mazel et al. 2018 Mazel et al. This content is distributed under the terms of the Creative Commons Attribution 4.0 International license.

10.1128/mSystems.00097-18.3 FIG S3 Theoretical expectation of the significance and strength of the correlation between microbiota composition and host phylogeny (i.e., phylosymbiosis) or host trait for varying microbiota-carrying capacities (i.e., the number of individual microbes in each host). The correlation between microbiota composition and host phylogeny (i.e., phylosymbiosis) is measured with two methods (the dendrogram-based method and the Mantel method [yellow and blue curves and boxplots, respectively]), while the correlation between microbiota composition and host trait is measured with a Mantel test (green curve and boxplots). Note that because microbiota have been directly filtered with the host trait, we expect the correlation between microbiota composition and this host trait to be higher than with host phylogeny (i.e., phylosymbiosis approaches), which imperfectly reflects the host trait. For phylosymbiosis (dendrogram-based and Mantel methods), the plots depict the relationship between the proportion of phylosymbiosis (i.e., the proportion of simulations where the phylosymbiosis metric was significantly different from null expectations, *y* axis, top plots) or the strength of the phylosymbiosis as a quantified by Pearson’s *r* or normalized Robinson-Foulds metric (*y* axis, bottom plots) and the phylogenetic signal of the host trait that filters microbes to assemble microbiotas (*x* axis). Note that Pearson’s *r* and the normalized Robinson-Foulds metric should be interpreted differently with respect to the strength of phylosymbiosis; normalized Robinson-Foulds metric values close to 0 indicate strong phylosymbiosis, while Pearson’s *r* values near 1 indicate strong phylosymbiosis. For the correlation between microbiota composition and host trait (green curves and boxplots), the plots depict the relationship between the proportion of significant Mantel tests (*y* axis, top plots) or the strength of the signal as a quantified by Pearson’s *r* (*y* axis, bottom plots) and the phylogenetic signal of the host trait that filters microbes to assemble microbiotas (*x* axis). The carrying capacity (i.e., number of individual microbes in each host) increases from the left to right panels. The simulated ecological filtering strength is set to 0.5 (i.e., beta.env = 0.5). These results are based on 1,000 simulation sets, with each set containing 150 microbial species and 25 host species. Download FIG S3, EPS file, 1.6 MB. Copyright © 2018 Mazel et al. 2018 Mazel et al. This content is distributed under the terms of the Creative Commons Attribution 4.0 International license.

10.1128/mSystems.00097-18.4 FIG S4 Theoretical expectation of the significance and strength of the correlation between microbiota composition and host phylogeny (i.e., phylosymbiosis) or host trait for varying numbers of host species. The correlation between microbiota composition and host phylogeny (i.e., phylosymbiosis) is measured with two methods (the dendrogram-based method and the Mantel method [yellow and blue curves and boxplots, respectively]), while the correlation between microbiota composition and host trait is measured with a Mantel test (green curve and boxplots). Note that because microbiota have been directly filtered with the host trait, we expect the correlation between microbiota composition and this host trait to be higher than with host phylogeny (i.e., phylosymbiosis approaches), which imperfectly reflects the host trait. For phylosymbiosis (dendrogram-based and Mantel methods), the plots depict the relationship between the proportion of phylosymbiosis (i.e., the proportion of simulations where the phylosymbiosis metric was significantly different from null expectations, *y* axis, top plots) or the strength of the phylosymbiosis as quantified by Pearson’s *r* or the normalized Robinson-Foulds metric (*y* axis, bottom plots) and the phylogenetic signal of the host trait that filters microbes to assemble microbiotas (*x* axis). Note that Pearson’s *r* and the normalized Robinson-Foulds metric should be interpreted differently with respect to the strength of phylosymbiosis; normalized Robinson-Foulds metric values close to 0 indicate strong phylosymbiosis, while Pearson’s *r* values near 1 indicate strong phylosymbiosis. For the correlation between microbiota composition and host trait (green curves and boxplots), the plots depict the relationship between the proportion of significant Mantel tests (*y* axis, top plots) or the strength of the signal as a quantified by Pearson’s *r* (*y* axis, bottom plots) and the phylogenetic signal of the host trait that filters microbes to assemble microbiotas (*x* axis). The number of host species increases from the left to right panels. The simulated ecological filtering strength is set to 0.5 (i.e., beta.env = 0.5). These results are based on 1,000 simulation sets, with each set containing 150 microbial species. Download FIG S4, EPS file, 1.6 MB. Copyright © 2018 Mazel et al. 2018 Mazel et al. This content is distributed under the terms of the Creative Commons Attribution 4.0 International license.

10.1128/mSystems.00097-18.5 FIG S5 Theoretical expectation of the significance and strength of the correlation between microbiota composition and host phylogeny (i.e., phylosymbiosis) or host trait for varying numbers of microbial species. The correlation between microbiota composition and host phylogeny (i.e., phylosymbiosis) is measured with two methods (the dendrogram-based method and the Mantel method [yellow and blue curves and boxplots, respectively]), while the correlation between microbiota composition and host trait is measured with a Mantel test (green curve and boxplots). Note that because microbiota have been directly filtered with the host trait, we expect the correlation between microbiota composition and this host trait to be higher than with host phylogeny (i.e., phylosymbiosis approaches), which imperfectly reflects the host trait. For phylosymbiosis (dendrogram-based and Mantel methods), the plots depict the relationship between the proportion of phylosymbiosis (i.e., the proportion of simulations where the phylosymbiosis metric was significantly different from null expectations, *y* axis, top plots) or the strength of the phylosymbiosis as a quantified by Pearson’s *r* or the normalized Robinson-Foulds metric (*y* axis, bottom plots) and the phylogenetic signal of the host trait that filters microbes to assemble microbiotas (*x* axis). Note that Pearson’s *r* and the normalized Robinson-Foulds metric should be interpreted differently with respect to the strength of phylosymbiosis: normalized Robinson-Foulds metric values close to 0 indicate strong phylosymbiosis, while Pearson’s *r* values near 1 indicate strong phylosymbiosis. For the correlation between microbiota composition and host trait (green curves and boxplots), the plots depict the relationship between the proportion of significant Mantel tests (*y* axis, top plots) or the strength of the signal as a quantified by Pearson’s *r* (*y* axis, bottom plots) and the phylogenetic signal of the host trait that filters microbes to assemble microbiotas (*x* axis). The number of microbial species decreases from the left to right panels. The simulated ecological filtering strength is set to 0.5 (i.e., beta.env = 0.5). These results are based on 1,000 simulation sets, with each set containing 25 host species. Download FIG S5, EPS file, 1.5 MB. Copyright © 2018 Mazel et al. 2018 Mazel et al. This content is distributed under the terms of the Creative Commons Attribution 4.0 International license.

10.1128/mSystems.00097-18.6 FIG S6 Theoretical expectation of the significance and strength of the correlation between microbiota composition and host phylogeny (i.e., phylosymbiosis) or host trait for varying numbers of colonization steps. The correlation between microbiota composition and host phylogeny (i.e., phylosymbiosis) is measured with two methods (the dendrogram-based method and the Mantel method [yellow and blue curves and boxplots, respectively]), while the correlation between microbiota composition and host trait is measured with a Mantel test (green curve and boxplots). Note that because microbiota have been directly filtered with the host trait, we expect the correlation between microbiota composition and this host trait to be higher than with host phylogeny (i.e., phylosymbiosis approaches), which imperfectly reflects the host trait. For phylosymbiosis (dendrogram-based and Mantel methods), the plots depict the relationship between the proportion of phylosymbiosis (i.e., the proportion of simulations where the phylosymbiosis metric was significantly different from null expectations, *y* axis, top plots) or the strength of the phylosymbiosis as a quantified by Pearson’s *r* or the normalized Robinson-Foulds metric (*y* axis, bottom plots) and the phylogenetic signal of the host trait that filters microbes to assemble microbiotas (*x* axis). Note that Pearson’s *r* and the normalized Robinson-Foulds metric should be interpreted differently with respect to the strength of phylosymbiosis; normalized Robinson-Foulds metric values close to 0 indicate strong phylosymbiosis, while Pearson’s *r* values near 1 indicate strong phylosymbiosis. For the correlation between microbiota composition and host trait (green curves and boxplots), the plots depict the relationship between the proportion of significant Mantel tests (*y* axis, top plots) or the strength of the signal as a quantified by Pearson’s *r* (*y* axis, bottom plots) and the phylogenetic signal of the host trait that filters microbes to assemble microbiotas (*x* axis). The number of colonization steps used in simulations (see Materials and Methods) increases from the left to right panels. The simulated ecological filtering strength is set to 0.5 (i.e., beta.env = 0.5). These results are based on 1,000 simulation sets, with each set containing 25 host species and 150 microbial species. Download FIG S6, EPS file, 1.2 MB. Copyright © 2018 Mazel et al. 2018 Mazel et al. This content is distributed under the terms of the Creative Commons Attribution 4.0 International license.

The third set of simulated microbiotas were deterministically assembled according to filtering by multiple host traits. We find that adding host filtering traits generally increases the significance (Fig. 2B) and strength (Fig. 3B) of phylosymbiosis. To test for the effect of microbe-microbe interactions in shaping phylosymbiosis, we added competition between microbes to our simulation scheme, in addition to the filtering process. Overall, we observe that microbe-microbe competition had only minor effects on our results, and our conclusions remain unchanged (Fig. 2C and 3A).

### Empirical evidence of phylosymbiosis.

**FIG 4:**
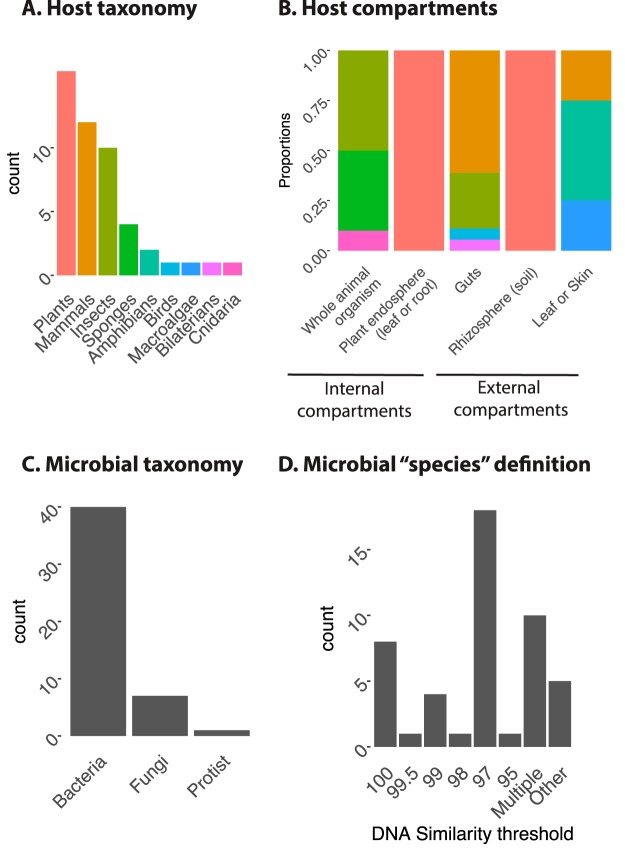
Overview of the studies included in the meta-analysis. Summary of the host and microbe information from the literature review (*n =* 48 studies), namely, the host (A) and microbial (C) taxonomic coverage, host compartments (B), and definition of microbial units (D).

**FIG 5:**
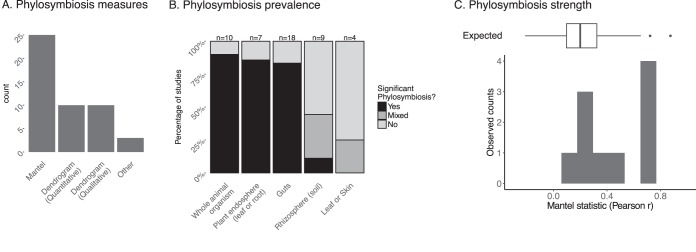
Measuring phylosymbiosis in nature. (A) Summary of tests used to measure phylosymbiosis in the literature review (*n* = 48 studies). See Table S1 in the supplemental material for details. (B) Prevalence of significant phylosymbiosis pattern by host compartment. (C) Phylosymbiosis strength distribution across studies that used Mantel tests as measured by Pearson’s *r*. “Expected” is the ecological filtering expectation from simulations corresponding to 25 host species, 150 microbial units, and moderate-to-strong phylogenetic signals of the host trait (Blomberg K, {0.5, 1.5}).

In order to compare theoretical expectations to observed patterns, we reviewed the strength and prevalence of phylosymbiosis in nature. We assembled a set of 36 articles that assess the correlation between host phylogeny and microbiota composition (see Table S1 and references given in the supplemental material for the complete list of studies). We included only those studies that explicitly used a host phylogenetic tree (not host taxonomy alone) and that included at least 4 taxa. In these 36 articles, 48 phylosymbiosis tests were performed in total, as some studies included multiple phylosymbiosis tests (e.g., one test for bacteria and one for fungi with the same host taxa; see Table S1). These studies encompass a wide range of plant and animal clades (Fig. 4A and B and Table S1) and various host compartments (gut, skin, leaves, root endosphere, rhizosphere) (Fig. 4B). Most of the studied microbiotas are composed of bacteria and archaea, with some studies focusing on fungi, notably for plants (Fig. 4C). Microbiota compositions are determined by defining microbial units from sequencing data, usually by grouping reads into operational taxonomic units (OTUs) according to a DNA similarity threshold (often 97% for the 16S rRNA gene, a rough proxy for bacterial species). Some studies measured phylosymbiosis by determining community compositions using multiple similarity thresholds (Fig. 4D). Approximately 52% of all studies used the Mantel approach, and ∼40% used the dendrogram-based approach (Fig. 5A). The dendrogram-based approach is used quantitatively (e.g., for measuring and testing the significance of the normalized Robinson-Foulds metric) or qualitatively (e.g., for visually assessing congruence between the dendrograms) in equal proportions. Only a small fraction of studies used other statistical methods (∼7% of all studies).

10.1128/mSystems.00097-18.9 TABLE S1 Studies used in the meta-analysis of phylosymbiosis prevalence in nature. The table presents a list of studies measuring phylosymbiosis alongside the taxonomic position, number, and taxonomic rank of host species, microbial taxa, and unit definition (i.e., the DNA similarity threshold used to define OTUs), the approach used to measure phylosymbiosis, the use of cofactors or intrahost species variability, and the results found. Download Table S1, XLSX file, 0.0 MB. Copyright © 2018 Mazel et al. 2018 Mazel et al. This content is distributed under the terms of the Creative Commons Attribution 4.0 International license.

Overall, phylosymbiosis is widespread in nature, being observed in the plant root endosphere, in insect and mammalian guts, and in sponges (Fig. 4 to 5). It is observed at various host phylogenetic scales (from subspecies to very large clades, such as bilateria) (Table S1). However, this pattern is not universal; it is, for example, absent in bird guts and plant rhizospheres (Table S1). Internal microbiotas (such as gut or plant endosphere) tend to harbor phylosymbiosis patterns much more frequently than external microbiotas (e.g., rhizospheres or animal skin) (Fig. 5B). Phylosymbiosis also varies greatly in strength, from 0.11 to 0.75 as measured by Pearson’s *r*, often used in the Mantel approach (Fig. 5C). In some cases, the strength of observed phylosymbiosis is higher than ecological expectations (compare the observed versus expected Pearson’s *r* distributions in Fig. 5C). Importantly, the resolution used to define microbial units seems to have an impact on the strength and prevalence of phylosymbiosis, as shown by the few studies that use multiple DNA similarity thresholds to delineate these units and measure phylosymbiosis (Table S1). For example, Groussin et al. (18) detected strong phylosymbiosis in the mammalian gut microbiota using ∼3% as the similarity threshold to define OTUs (*r*^2^ ∼ 0.4) but measured very weak signal (*r*^2^ < 0.05) when considering similarity threshold higher than 9% (91% OTUs).

The estimation of the genuine prevalence and strength of phylosymbiosis in nature is hampered by several factors. First, different studies often use different methods to quantify phylosymbiosis. Furthermore, methodological inconsistencies in data collection and experimental measurements across studies limit our ability to confidently estimate the strength and universality of phylosymbiosis. In particular, some studies control for host traits that are known to influence microbiota composition, such as diet for gut microbiota (10), using controlled lab conditions. Other studies use statistical methods to control for confounding host traits (Table S1), but the majority of studies do not perform any such controls (Table S1). Finally, intraspecific variability is sometimes ignored (i.e., sampling only one individual per host species), sometimes pooled (either during library preparation or *in silico*), or taken into account by considering a null distance for the host phylogenetic distances between individuals of the same species (Table S1). Despite these limitations, phylosymbiosis appears to exist in major clades across the tree of life, motivating the development of new approaches to fully understand its origins and complexity.

## DISCUSSION

### How to measure phylosymbiosis.

Our literature review reveals that phylosymbiosis has been measured in a variety of ways, controlling or not for confounding host traits and intraspecific variability. We offer several recommendations to unify phylosymbiosis measurements and more accurately detect it.

### (i) How to define microbial units.

Microbial units are usually defined using the somewhat arbitrary threshold of 97% DNA similarity. With the development of new efficient algorithms (21) that detect and remove nonbiological reads, like chimeras, microbial units can now be defined at 100% similarity, at the sequence variant level, without binning into operational taxonomic units (OTUs). We believe that this relatively fine-scale definition of microbial units will enable detection of more fine-scale phylosymbiosis because it will better differentiate between closely related microbes that might occur in closely related hosts. However, several studies have found that phylosymbiosis strength and significance can vary along the phylogenetic (or 16S rRNA similarity) scale (22) and is not necessarily maximal at the finest taxonomic resolution (18, 23). As a consequence, we suggest that multiple phylogenetic (or 16S rRNA similarity) thresholds (24, 25) should be used to measure phylosymbiosis (starting with the sequence variants as the finest taxonomic resolution) and that phylosymbiosis strength should be analyzed and reported along this resolution gradient (18).

### (ii) Which beta-diversity metric to use.

A number of different metrics have been used so far to detect phylosymbiosis. We found that use of the weighted UniFrac metric, which considers abundance, yielded a slightly stronger phylosymbiosis signal than unweighted UniFrac analysis, which does not consider abundance. This is in agreement with our simulation model; simply by chance, a given individual microbe may colonize a host, even if it is not the best-adapted microbe for a given host trait. However, these events will be rare and the majority of individuals colonizing a host are the best adapted ones. As such, abundance weighted metrics (such as weighted UniFrac or Bray-Curtis metrics) will have more power to detect the filtering process. Although we found that the differences in phylosymbiosis detection between beta-diversity metrics were not striking, we suggest that future tests of phylosymbiosis should use and report the results for multiple metrics (both abundance-based metrics and presence/absence metrics) to enable better assessment of the robustness of the results.

### (iii) Which phylosymbiosis statistic to use.

We suggest that future studies documenting phylosymbiosis should be backed by statistical validation, which has been lacking in a number of past studies. Of course, visual inspections of congruencies between host phylogeny and microbiota compositions will continue to be important for assessing the biological significance of the patterns and gaining additional insight into these data (akin to nonmetric multidimensional scaling [NMDS] visualizations of community dissimilarity), but they should always be accompanied by statistical investigations. The two most commonly employed statistical procedures are the Mantel approach and the dendrogram-based approach. Our results show that the Mantel test is more powerful than dendrogram-based approaches at detecting ecological filtering. The dendrogram-based approach starts by building a community dendrogram from the raw beta-diversity values, usually using a hierarchical clustering approach such as the unweighted pair group method using average linkages (UPGMA). However, this step might not be necessary because there is no *a priori* reason (or, at least, no empirical evidence so far) to think that microbiota compositions relate to each other in a hierarchical manner. More importantly, this transformation into a dendrogram comes at a cost of data loss because distances between microbiota compositions based on the dendrograms and raw (true) distances are never perfectly correlated. This data loss might explain why the dendrogram-based approach seems to have a reduced power in our simulations. However, we found that the 10 empirical phylosymbiosis tests carried out with this approach detected phylosymbiosis, whereas the Mantel approach detected phylosymbiosis in only 30% of tests. While this result appears in contradiction with our theoretical findings, it is likely explained by the fact that most of the dendrogram-based tests used gut microbiomes, a host compartment that is known to harbor strong phylosymbiosis. In summary, our results suggest that the Mantel approach is preferable to the dendrogram-based approach to measure phylosymbiosis, although the validity of the Mantel test is also debated (26, 27). In the future, other distance-based approaches could be considered, for example Procrustes analysis (28) and multiple regression analysis on distance matrices (29), which have the advantage of allowing the inclusion of other host traits (see below).

### (iv) How to handle intrahost species variability.

In this paper, we focused our analyses on phylosymbiosis at the interspecific level, using only 1 individual per host species in our simulations. However, phylosymbiosis may also occur within host species, e.g., between populations. There is some empirical evidence that within-species phylogenetic distances are correlated with microbiota compositions (30), but almost all studies so far have defined and investigated phylosymbiosis at the interspecific level. In these studies, when multiple individuals per species were included, it was sometimes assumed that all individuals were replicates of the same phylogenetic bins, namely, that they have null phylogenetic distances between them. However, because microbiota dissimilarities within species generally tend to be smaller than dissimilarities between species, reducing all individuals within a species to the same phylogenetic bin can create a spurious phylosymbiosis when it is measured with the Mantel test, even in the absence of interspecific phylosymbiosis (31) (see also Fig. S7 in the supplemental material, which describes this phenomenon). In order to tease apart the effects of intra- versus interspecific variations in microbiota compositions, and in the absence of data on intraspecific genetic distances, we suggest conducting two separate tests. First, a permutational multivariate analysis of variance (PERMANOVA) (32) could be used to detect the intra- versus interspecific patterns, i.e., the fact that individuals within a species will harbor more similar microbiota than individuals from two different species. Second, intraspecific microbiota could be pooled to produce a unique microbiota for each species, which can then be used to detect phylosymbiosis at the interspecific level, for example using a Mantel test.

10.1128/mSystems.00097-18.7 FIG S7 The effects of intra- versus interspecific microbiota comparisons on phylosymbiosis. The figure depicts a theoretical case where intraspecific comparisons (blue points) and interspecific comparisons (red points) are included in a Mantel test. Each point represents a comparison between two samples. Here, intraspecific phylogenetic distances (*x* coordinates of the blue points) are set to zero because there is no information on genetic distances between individuals within a species (as is often the case). While there is no signal at the interspecific scale (i.e., the red line fit is flat), phylosymbiosis appears if all comparisons are included (grey line fit). However, there is no *a priori* effect of host phylogenetic distances but only an effect of host species identities (i.e., intraspecific beta-diversities [*y* coordinates of blue points] are lower than interspecific beta-diversities [*y* coordinates of red points]). Download FIG S7, EPS file, 0.1 MB. Copyright © 2018 Mazel et al. 2018 Mazel et al. This content is distributed under the terms of the Creative Commons Attribution 4.0 International license.

### (v) How to handle the impact of other host traits.

Using simulations, we showed that phylosymbiosis can simply reflect the effect of filtering by a phylogenetically conserved host trait (e.g., diet or gut pH) on the assembly of microbiotas. If this host trait is known and measured across host species, it can be directly used to explain microbiota compositions, and the effect of host phylogeny could be measured on the remaining unexplained variability in microbiota compositions. If this additional variability is related to host phylogeny, it may represent the effect of another unmeasured (but phylogenetically conserved) host trait. Teasing apart the effects of the measured host traits and host phylogeny can be done by conducting experiments under controlled conditions for host trait (e.g., diet [10]) or by incorporating the different factors directly into the statistical analysis as explanatory variables, using variance partitioning (18, 20). For example, the regression on multiple distance matrices (29) (function MRN in the R package ecodist [33]) allows for multiple factors to be considered simultaneously, which represents another reason for favoring Mantel-like approaches over the dendrogram-based approaches to measure phylosymbiosis.

### Ecological filtering can produce phylosymbiosis in simulated data sets.

The detection of the phylosymbiosis of microbial compositions has recently gained much attention (Table S1). However, the mechanisms responsible for this intriguing pattern remain controversial (11, 13, 15). While simulation studies are, by definition, limited to a restricted set of an infinite parameter space, the simplicity of our approach allowed us to test an important aspect of host-associated microbiota evolution. We provide, to our knowledge, the first direct and quantitative evidence that simple ecological filtering can generate phylosymbiosis. This happens when the host trait that directly filters microbes also harbors some degree of phylogenetic signal, i.e., when host trait values are more similar between closely related species than between distantly related species. This means that phylosymbiosis alone cannot be taken as a proof of an intimate and long-term coevolution between the host and the microbiota. While this point was acknowledged in previous reports (11, 15, 34), we explicitly show it using simulations. We note here that our simulations did not explore intraspecific variability compared to interspecific variability. However, we see no reasons why our results should not also apply to within-host species comparisons. In summary, our simulations demonstrate that, in theory, ecological filtering can produce phylosymbiosis. The next natural step is then to test whether these theoretical expectations match the patterns observed in nature.

### Comparing theory and data.

**(i) Empirical patterns of phylosymbiosis can be explained by simple ecological filtering.** Phylosymbiosis seems widespread in nature but is clearly not universal. Even if the prevalence of phylosymbiosis that we estimated here is likely to be overestimated because of publication bias (i.e., negative results tend to be less published [35]), we show that observed phylosymbiosis prevalence (Fig. 5B) and especially strength (Fig. 5C) match ecological filtering predictions. This finding demonstrates that phylosymbiosis observed in nature can be explained by filtering processes based on host traits that have moderate phylogenetic signals. More precisely, we suggest that ecological filtering represents a parsimonious and sufficient explanation for most phylosymbiosis patterns observed in nature. Of course, our work, as a simulation experiment, provides theoretical expectations only as to when phylosymbiosis may occur under our simulated filtering scenario. Given similar observed and expected patterns, we can suggest (but not directly conclude) that the set of hypotheses that we used to build our simulations are sufficient (but not necessary) to explain the pattern observed in nature without invoking more-complex processes, such as codiversification. As such, our quantitative ecological filtering expectations represent a needed step forward. We note that our expectations depend on the choice of simulation parameters and that we did not directly measure these parameters in nature. This is a particularly important caveat, especially regarding the strength of phylogenetic signal of the host filtering trait; we do not know where host traits fall on the *x* axis of Fig. 2 to 3 in nature, though it is generally accepted that very high Blomberg K values (≥1) are rare and that most traits harbor Blomberg K values of ≤1 (36). Of course, this assumes that previous measurements of phylogenetic signal on “classical” host traits often considered in macroevolution (e.g., body mass) are representative of the phylogenetic signal of the traits that directly filter microbes. Testing the validity of this hypothesis is beyond the scope of this study but represents an interesting avenue for future work, especially to test whether important host traits (e.g., gut pH or morphology) harbor stronger phylogenetic signal than the classical traits studied in macroevolution.

In summary, our results suggest that the majority of phylosymbiosis patterns detected in the literature (Fig. 5) are compatible with simple ecological filtering. This conclusion holds true irrespective of the method (Mantel or dendrogram based) or beta-diversity metric used. Importantly, we are not claiming that an ecological filter applies as a present-day process only. Filtering effects have likely emerged and evolved over long evolutionary times. Hosts evolved microbial habitats with different properties (e.g., different pHs, gut/tissue structures, diets), and it is likely that different microbes have specialized over long time scales to these different habitats through adaptive evolution (37). However, coevolution, defined as the “reciprocal genetic change in interacting species, owing to natural selection imposed by each on the other” (38), or even codiversification processes are not necessary to explain this pattern. Rather, the host surface could be compared to an inert, but changing, habitat that simply filters microbes based on their traits. We are well aware that microbes also directly modify the host environment (e.g., fermentative gut microbes can reduce gut pH) and that this environment is not “inert,” but our aim here is to test the validity of this simple explanation for phylosymbiosis. Ecological filtering expectations represent a starting point from which new hypotheses can be generated, especially when mismatches emerge between theory and data. For example, our literature review highlights a deviation in phylosymbiosis signal and strength when comparing internal versus external microbiotas, suggesting that these broad categories of microbiota are assembled via different mechanisms.

**(ii) Deviations from theoretical expectations.** Empirical evidence suggests that, for some types of microbiotas, phylosymbiosis can be relatively strong, such as in the case of mammalian gut microbiotas (18) (Fig. 5C). A strict host filtering process could theoretically produce such a strong signal, but the amount of phylogenetic signal needed to produce such a pattern is much greater than those of typical host traits (Blomberg K, ≫1) (Fig. 5C). Then, how can we reconcile these seemingly contradictory findings? Either traits associated with the gut harbor stronger phylogenetic signals than average traits, or other processes, such as codiversification or even coevolution, are at play to generate these strong phylosymbiosis signals. Indeed, codiversification has been shown to reinforce phylosymbiosis (18), so that, coupled with ecological filtering, it could produce stronger phylosymbiosis than expected. To be able to further test the codiversification hypothesis, we encourage researchers to use tools that are especially dedicated for this purpose. For example, the amalgamated likelihood estimation (ALE) algorithm (39) can detect cospeciation and host shift events along the microbial phylogenetic tree by reconciling the symbiont phylogenetic tree with the host tree using a probabilistic model of cospeciation, host swapping, intrahost speciation, and extinction of symbionts within the host tree. Coevolution between specific microbes and their hosts may also contribute to phylosymbiosis patterns and strengthen ecological filtering. Overall, ecological filtering, codiversification, and coevolution are not mutually exclusive processes that can produce phylosymbiosis. In conclusion, while deviations from our ecological filtering expectations cannot be readily linked to a given process, they offer the opportunity to explore and formulate new hypotheses about microbiota assembly and evolution.

### Future directions.

**(i) Heterogeneity of phylosymbiosis.** In this paper, we considered a measure of phylosymbiosis for the overall host phylogenetic tree. However, it is increasingly recognized that macroevolutionary patterns can vary across the phylogenetic tree (40), even in the case of phylosymbiosis (18, 41, 42). These variations across the host tree could, for example, reflect an accelerated evolution of the host traits that filter microbiotas or accelerated allopatric speciation events on some parts of the host tree. To detect such patterns, future studies could measure phylosymbiosis on multiple subclades of hosts by splitting the overall host phylogeny (41) or use a node-based measure of phylosymbiosis (18).

**(ii) Toward explicit models of microbiota macroevolution.** The seemingly high prevalence of phylosymbiosis is stimulating research on the micro- and macroevolution of host-associated microbiotas (43, 44), but more-fundamental questions concerning the tempo and mode of host-associated microbiota macroevolution remain unanswered. For example, where in the host tree of life can we detect shifts in rates of microbiota evolution? Are these shifts related to evolutionary transitions of host traits, such as diet? Are different groups of microbes following different macroevolutionary dynamics? Does microbiota evolution occur early in the history of a host clade, as it is often expected for adaptive radiations (45, 46)? Is this evolution pulsed or continuous? To answer these questions, we need to go beyond measuring crude phylosymbiosis. Indeed, as a measure of phylogenetic signal, phylosymbiosis has limited power to differentiate between alternative macroevolutionary models (40). While some authors have already tried to measure heterogeneous signal of phylosymbiosis across the host tree (18, 41), we believe that the development of more-explicit (and mechanistic) evolutionary models, as is currently implemented for macroevolutionary studies (40, 47) and even in microbiome studies (43, 44), is needed and holds great promise. For example, comparing the fits of simple drift models (such as Brownian motion-like models) to the fit of multirate or multioptimum models (such as Ornstein-Uhlenbeck-like models) could detect major evolutionary transitions (e.g., toward convergent phenotypes). Of course, microbiotas represent a very different type of host trait from the ones studied in macroevolution (37, 43, 44). The microbiota as a host trait is highly multidimensional, while more classical host traits, such as body mass, are relatively simple. While this impedes the direct use of existing, explicit macroevolutionary models to study microbiota, we suggest that, as in macroevolution, the use of more-mechanistic models of evolution will provide important insights into the mode and tempo of microbiota evolution.

## MATERIALS AND METHODS

### General approach.

The aim of our simulations is to test to what extent a simple ecological filtering process can produce patterns of phylosymbiosis. Our main hypothesis is that microbiota composition will exhibit phylosymbiosis if it is assembled via a host filtering process controlled by a host trait that itself harbors phylogenetic signal. To test this hypothesis, we simulated multiple microbiota sets under a filtering process controlled by a host trait and, for each set, varied the amount of phylogenetic signals in this host trait from no phylogenetic signal to a strong phylogenetic signal. Each set consisted of 250 unique simulations with identical parameter values and yielded an estimate of phylosymbiosis probability of detection. Each unique simulation consisted of (i) creating host and microbial species and traits, (ii) creating host-associated microbiotas based on known assembly rules, and then (iii) measuring the phylosymbiosis in the resulting microbiotas (Fig. 1).

### Step 1: host and microbe species pools.

Each unique simulation was based on a unique data set containing *n* hosts species and *m* microbial species or OTUs (we varied these numbers in different simulation sets; *m* = {75, 150} and *n* = {10, 25}). Different hosts harbored different trait values (e.g., gut pH), and different microbes harbored different host trait preference values (e.g., gut pH preference). We simulated these traits as follows: first, phylogenies for both host and microbes were simulated with a pure birth model and rescaled the height of the tree to 1 so that the heights were comparable across simulations; second, we independently simulated one single continuous host trait (e.g., gut pH) and one single continuous microbial trait preference (e.g., pH preference). In order to vary the degree of phylogenetic signal harbored by the host trait, we used the delta model of trait evolution (7). The delta model is a time-dependent model of trait evolution; when delta is greater than 1, recent evolution has been relatively fast, but if delta is less than 1, recent evolution has been comparatively slow. To implement it, we rescaled the tree branch raising all node depths to an estimated power (delta) and then ran a classic Brownian motion model on this rescaled tree. We used five values of delta {0.01, 0.1, 1, 10, 1,000} for host trait and one value {1} for the microbial trait and recorded the corresponding phylogenetic signals of the traits using the Blomberg K index (36). An interesting avenue for future studies would be to also vary the model of microbial trait evolution (e.g., varying delta) and study to what extent this impacts the phylogenetic resolution of microbial units at which phylosymbiosis is best detected. Finally, we rescaled the microbial traits so that they varied within the range of host trait values. We also varied the number of traits used to assemble microbiota; in addition to the single trait used in the main text, we used 2 and 4 traits (traits followed the same evolutionary model).

### Step 2: microbiota assembly.

For each unique data set and associated simulation independently, we assembled *n* host microbiotas (one for each host) based on simple ecological filtering using the R package VirtualCom (48), which simulates models of ecological community assembly. Briefly, the individual-based simulation model assumes that the probability of a given microbial individual to colonize a given host depends linearly on the match between the host trait (e.g., gut pH) and microbial preferences for this host trait (e.g., pH preference of the microbes). In other words, a given microbe will be more likely to colonize a given host if the distance between its host trait preference (e.g., pH preference) and the host trait is small (see reference 48 for more details). In the main text results, the following parameters were used: a niche.breadth (value of standard deviation of the Gaussian distributions that describe the niches) of 0.5; a beta.env (value of the strength of the environmental filter) of 1, a beta.abun (value of the strength of the reproduction filter) of 0, a beta.comp (value of the strength of the competition filter) of 0, years equal to 20 (number of simulated time steps), and a K (carrying capacity, i.e., the maximum number of individuals in the community) of 150 (see reference 48 for more details). To test the robustness of our results to variation in these parameters, we carried out additional simulations varying beta.env, beta.abun (0.5 each), the number of years (30), and the number of individuals (300) (see Table S2 for details). It is important to note that our simulation workflow is meant to assemble microbiotas based only on filtering processes (i.e., there is no cospeciation or coevolution), and thus, it corresponds to the “ecological” case depicted by Moran and Sloan (13). As a negative control, we also simulated a “neutral” microbiota with no filtering process by setting beta.env to 0 and beta.abun to 1. We also conducted a simulation experiment where competition between microbes was added, setting the beta.comp parameter to 0.25 and beta.env and beta.abun to 0.5 and 0.25, respectively.

10.1128/mSystems.00097-18.10 TABLE S2 List of simulation parameters and associated figures. Each column depicts a simulation set, a corresponding figure, and its associated simulation parameters. Details on the simulation parameters are given in Materials and Methods, and the R code is available at https://github.com/FloMazel/Phylosymbiosis-Ecological-model. Download Table S2, PDF file, 0.0 MB. Copyright © 2018 Mazel et al. 2018 Mazel et al. This content is distributed under the terms of the Creative Commons Attribution 4.0 International license.

### Step 3: measure of phylosymbiosis.

For each unique simulation, we measured the phylosymbiosis of the resulting set of microbiota using four measures of beta diversity (Jaccard, Bray-Curtis, weighted UniFrac, and unweighted UniFrac) and two approaches to measure correlation between host phylogeny and microbiota composition: the dendrogram approach (9, 10) and the Mantel approach (20). The dendrogram approach first produces a hierarchical dendrogram depicting microbiota beta diversity using the UPGMA. It then quantifies the match between the microbial dendrogram and the host phylogeny using the normalized Robinson-Foulds metric. Significance is based on 999 permutations of host tip label phylogeny. Note that this is different from the original dendrogram-based approach that uses random phylogenies with the same numbers of tips as null expectations. However, the two null models gave nearly exactly the same results (Fig. S8), so we present only the tip-shuffling null model in the main text. The Mantel approach simply computes the Pearson correlation between host phylogenetic distance and microbial beta diversity. Significance is based on 999 permutations of the distance matrix. As a sanity test, we also measured to what extent the host trait used to assemble microbiota predicts microbiota compositions. To do so, we used the same procedure as the Mantel approach described above but using host trait distance instead of host phylogenetic distance. This test should yield maximum power estimates because it directly uses the host trait values used to assemble microbiota.

10.1128/mSystems.00097-18.8 FIG S8 Phylosymbiosis as measured by the dendrogram-based approach with an alternative null model. This figure is equivalent to Fig. 2A of the main text: the plots depict the relationship between the phylogenetic signal of the host trait that filters microbes to assemble microbiotas (simple ecological process) and the proportion of phylosymbiosis detected in the resulting microbiotas for a single host filtering trait. Here, two alternative null models are compared for assessing significance in the dendrogram-based approach: one model (“tip shuffling,” as in the main text) simply shuffles the tip labels of the host phylogenetic tree, while the second null model creates a set of new host trees with the same number of tips (“random trees”). The two models give very similar results and so are nearly indistinguishable. Note that multiple simulations have been grouped in bins of host trait phylogenetic signal (*x* axis) in order to compute the percentage of simulations that yielded phylosymbiosis. These results are based on 1,000 simulation sets in total, with each set containing 150 microbial species and 25 host species. Download FIG S8, EPS file, 0.2 MB. Copyright © 2018 Mazel et al. 2018 Mazel et al. This content is distributed under the terms of the Creative Commons Attribution 4.0 International license.

All analysis was carried out in R version 3.3.2 (49), with extensive use of the geiger (50), ape (51), phytools (52), GUniFrac (53), vegan (54), VirtualCom (48), ggplot2 (55), and phangorn (56) packages. Corresponding scripts are available at https://github.com/FloMazel/Phylosymbiosis-Ecological-model.
